# Comminuted supracondylar femoral fractures: a biomechanical analysis comparing the stability of medial versus lateral plating in axial loading

**DOI:** 10.1007/s11751-016-0268-0

**Published:** 2016-10-12

**Authors:** Nikolai Briffa, Raju Karthickeyan, Joshua Jacob, Arshad Khaleel

**Affiliations:** Trauma and Orthopaedic Department, Ashford and St. Peter’s Hospital NHS Trust, London, UK

**Keywords:** Supracondylar femur fracture, Medial versus lateral plating, Axial loading testing, Construct stability

## Abstract

The aim of this study was to compare the biomechanical properties of medial and lateral plating of a medially comminuted supracondylar femoral fracture. A supracondylar femoral fracture model comparing two fixation methods was tested cyclically in axial loading. One-centimetre supracondylar gap osteotomies were created in six synthetic femurs approximately 6 cm proximal to the knee joint. There were two constructs investigated: group 1 and group 2 were stabilized with an 8-hole LC-DCP, medially and laterally, respectively. Both construct groups were axially loaded. Global displacement (total length), wedge displacement, bending moment and strain were measured. Medial plating showed a significantly decreased displacement, bending moment and strain at the fracture site in axial loading. Medial plating of a comminuted supracondylar femur fracture is more stable than lateral plating.

## Introduction

Whilst distal supracondylar femoral fractures have been treated with skeletal traction historically and this has healed well, there were some morbidity and mortality [1]. Operative fixation is the gold standard currently. Several stabilization modalities are used: a single lateral buttress plate; a fixed-angled plate; an antegrade or retrograde intramedullary nail; a combination of medial and lateral plates; fine-wire circular external fixators, hybrid fixation; or even a primary total knee arthroplasty [2–8]. The ideal fixation, in the presence of comminution, is unconfirmed.

A biomechanical study was designed utilizing a comminuted supracondylar femoral fracture sawbone model. Two single plating fixation methods were tested cyclically in axial loading.

Our null hypothesis was that no mechanical difference exists between lateral plating and medial plating in this simulated supracondylar femur fracture with medial comminution.

## Materials and methods

Eight identical synthetic sawbone femurs (Synbone^®^) were utilized. These standard sawbones are made of polyurethane foam with a hollow canal. The femur is made of different densities of polyurethane at the epiphysis and diaphysis in order to simulate the native modulus of elasticity.

**Fig. 1:**
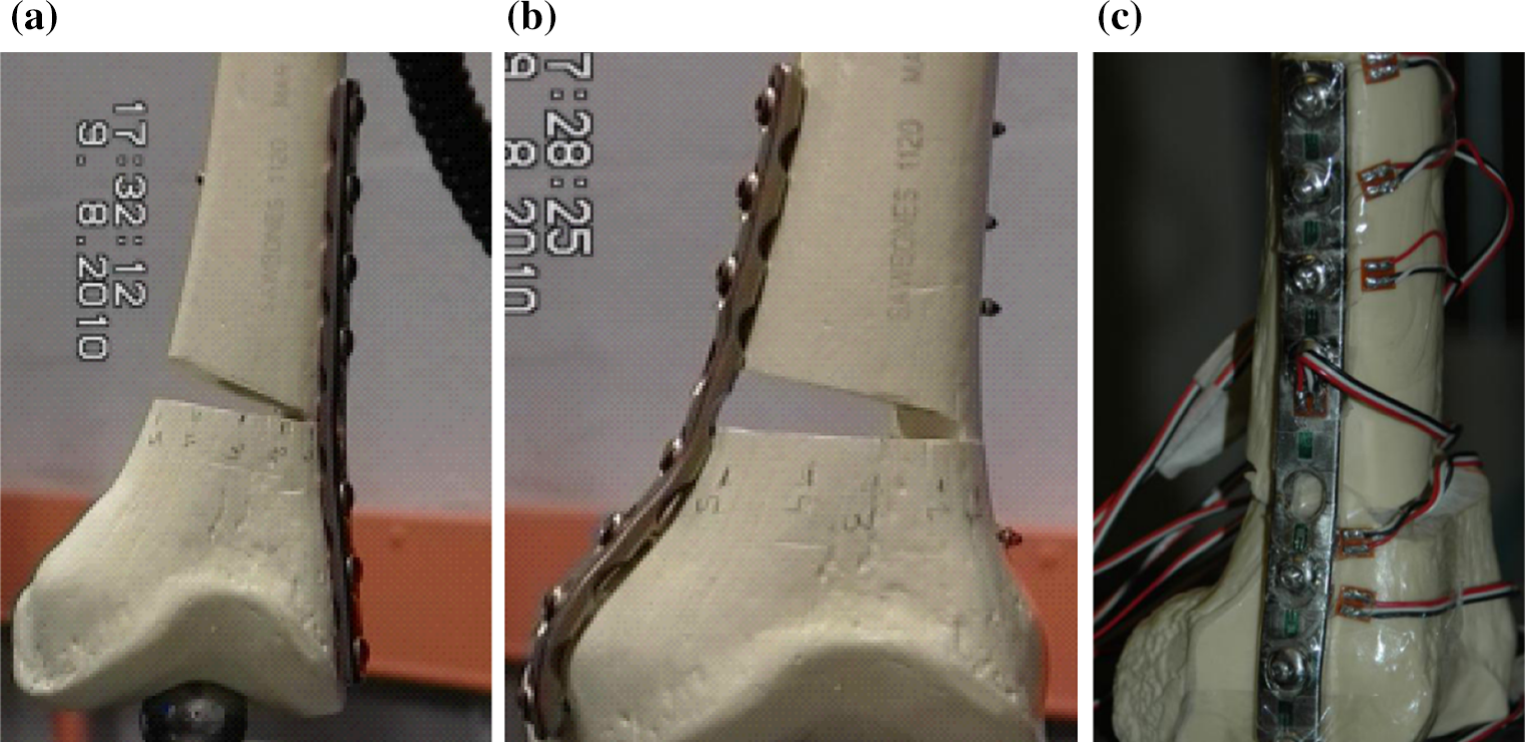
**a** Femur with distal lateral fixation. **b** Femur with distal medial fixation. **c** Position of strain gauges 1–5

A standardized fracture pattern simulating a supracondylar femoral fracture with medial comminution was created. This was achieved by creating a 1-cm supracondylar gap osteotomy in the eight synthetic femurs 4 cm proximal and parallel to the epicondylar axis as depicted in Fig. 1. This was to simulate medial metaphyseal comminution where contact between bone fragments is minimal.

**Fig. 2:**
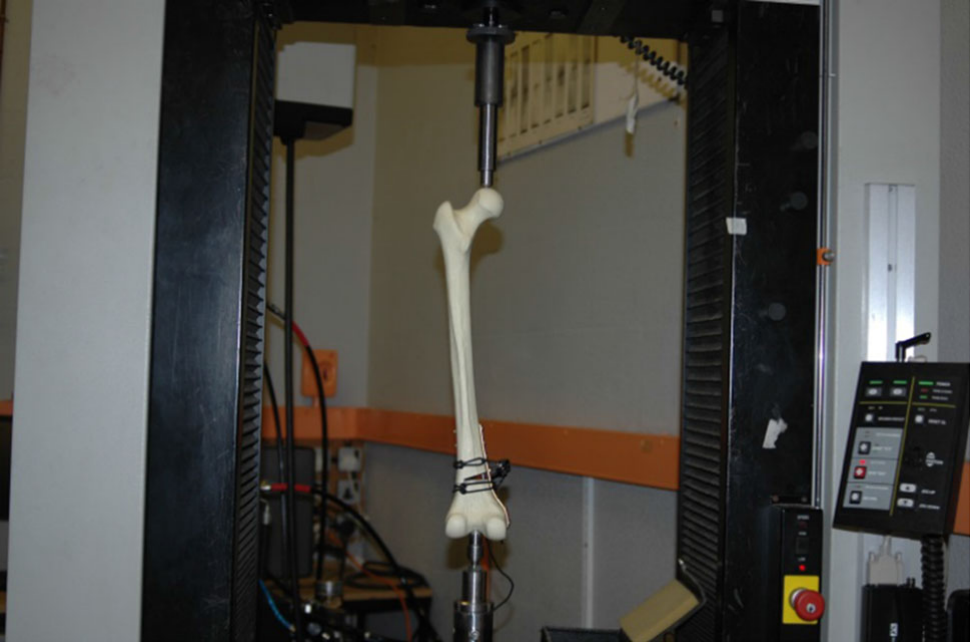
Femur specimen loaded in the Instron test machine simulating in vivo loading with the extensometer straddling the open face of the wedge gap

There were two groups. Group 1 (*n* = 4) had medial plate fixation and Group 2 (*n* = 4) had lateral plating, both with a contoured AO (Synthes) LC-DCP plate. One specimen from each group had five strain gauges placed on the fixing plate so that the strains in the plate could be recorded as a function of increasing load (Fig. 1c). Compressive loading was applied along the mechanical axis by an Instron^®^ 5500R (Model 1185) test machine (Fig. 2). This simulated the normal in vivo loading. Each of the eight specimens experienced three cycles of loading up to 500 N followed by unloading. Global displacement (total length), wedge displacement, bending moment and plate strain were recorded.

## Results

### Global displacements

**Fig. 3:**
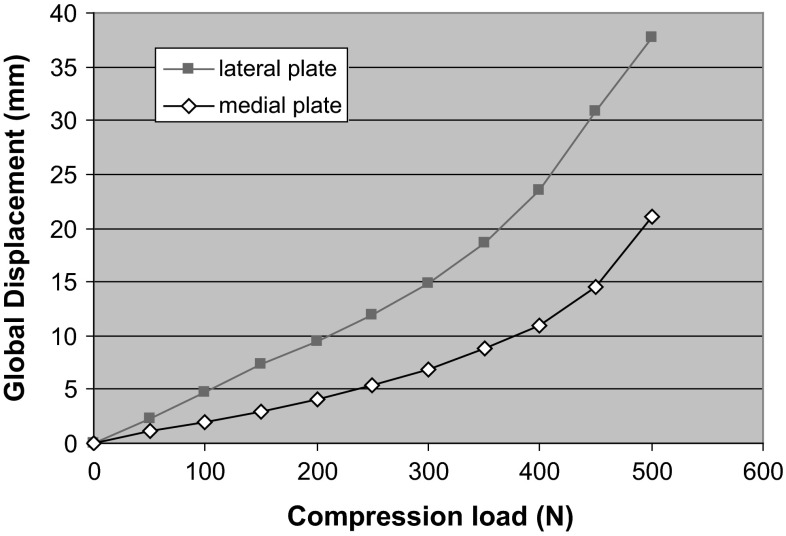
Lateral and medial plated global femur displacements as a function of load

**Fig. 4:**
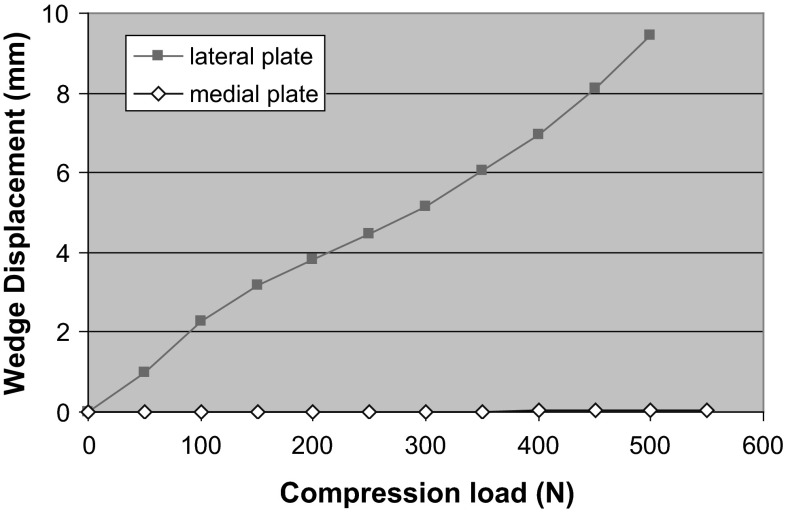
Lateral and medial plated wedge displacements as a function of load

Typical plots of crosshead displacement as a function of load for a lateral plated and a medial plated femur are shown in Fig. 3. The medially plated femur is a much stiffer construct and deforms less than the laterally plated femur as shown in Fig. 4.

### Wedge displacement

Typical plots of wedge displacement for both medial and lateral plates (Fig. 4) depict insignificant displacement on the medial side compared to the lateral construct.

### Plate strains

**Fig. 5:**
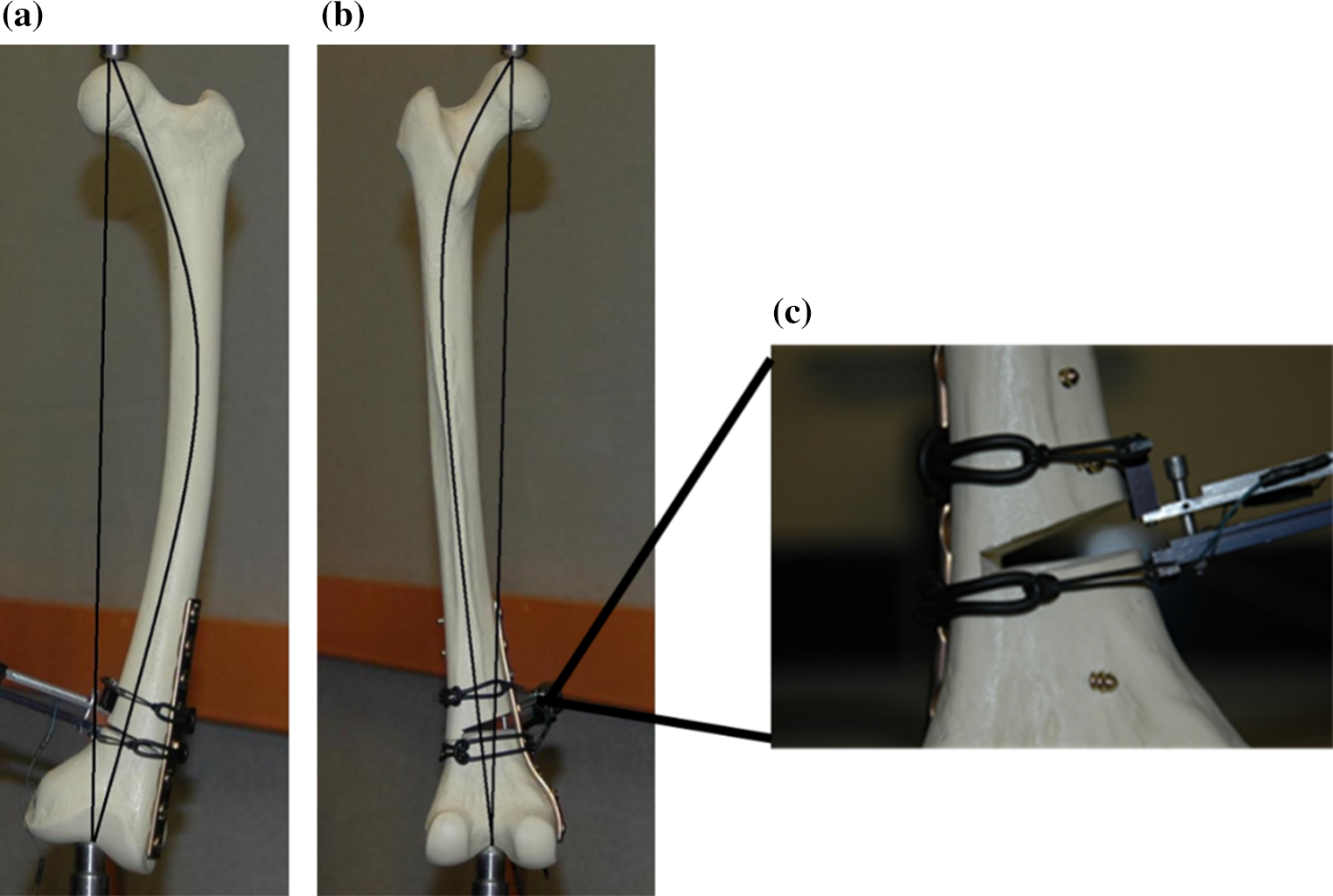
Deformed femur **a** lateral plate at 250 N, **b** medial plate at 500 N, **c** extensometer

**Fig. 6:**
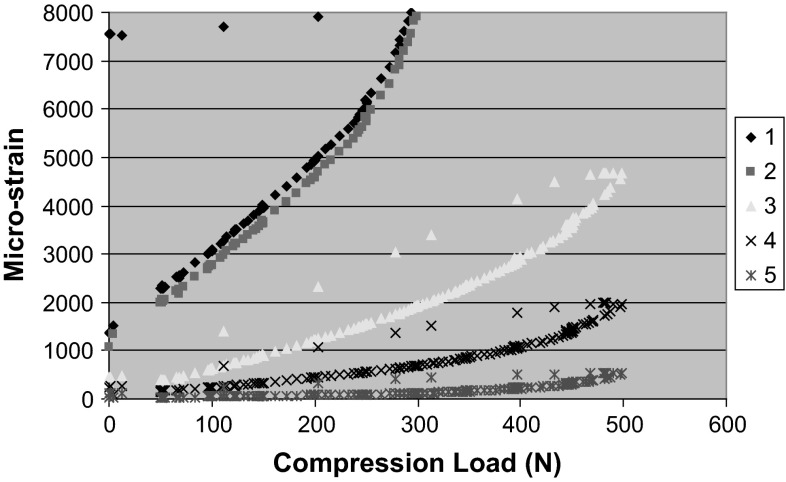
Strains recorded with lateral plating with legends 1–5 depict the various gauges along the femur. Unloading data of gauge 2 are not available due to malfunction

**Fig. 7:**
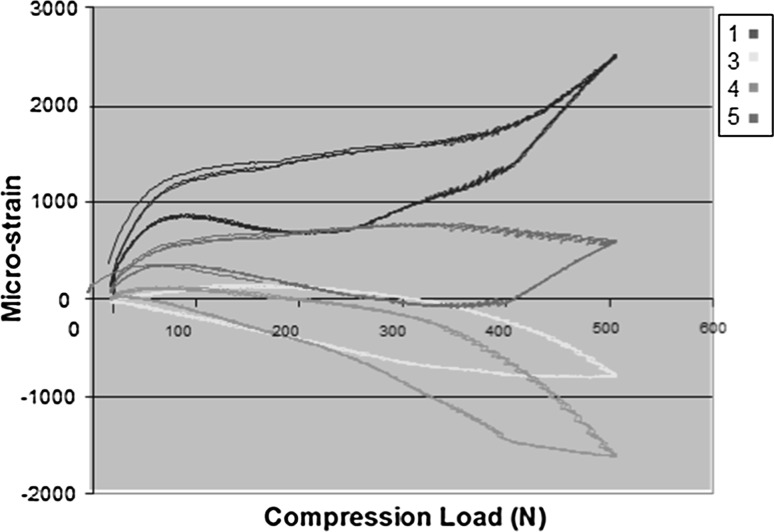
Strains recorded with medial plating with legends 1, 3, 4, 5 depict the various gauges along the femur. Data from gauge 2 are discarded due to malfunction on unloading

Strains were measured at five different locations for both lateral and medial plated models (Fig. 1c) during both loading and unloading. Plate strains varied considerably between medial and lateral plating. The loading curve follows the line of steepest gradient for all gauges. It can be seen that there is no variation between cycles. Gauge 2 was found to have failed and so data from it were not utilized. The strains along the medial plate–bone construct are of two orders of magnitude lower than the strains recorded with lateral plating (Figs. 5, 6, 7).

## Discussion

Open reduction and internal fixation is the treatment of choice for comminuted supracondylar fractures of the distal femur. Conventional plating, cable wiring, external fixators and, recently, low-profile contoured dynamic compression locking plating systems have been utilized [9–11]. New dedicated fixation systems merge conventional and locking screw technology, allowing the surgeon to achieve angular stability and compression in tension with a small footprint (minimally invasive plate osteosynthesis technique). Despite this, the non-union rate is considerable. It was proposed that this was due to a fall in strain occurring within the zone of injury as bone healing progressed [9]. To overcome this, the dynamic locking screws pin–sleeve design that combines locking technology with dynamic motion was introduced. The play between the sleeve and pin determines the amount of micro-motion induced in the fracture gap, decreasing the construct stiffness; this was thought a beneficial feature when bridging is the chosen method of fracture treatment [10]. Modern fracture stabilization aims for mechanical stability whilst protecting the biological environment of damaged bone and surrounding tissues, thereby optimizing the healing environment [12–15]. In supracondylar fractures of the femur which approach is best, direct lateral or medial?

This biomechanical study demonstrates that a plate placed medially is a more stable construct for this specific fracture simulation, namely medial comminution with the lateral cortex in contact to share load. These are conclusions drawn from an in vitro study, which emulates a clinical scenario. Anatomical reduction without undue biological disruption in metaphyseal comminuted fractures is rarely possible, leaving mechanical stability to be paramount. This has been shown to influence bony union positively [15]. Lateral plating alone supports the medial column indirectly and allows for a greater stress concentration on that side. Failure of fixation, through plate breakage, screw pull out and varus collapse, is a potential complication [16] and are more likely with lateral than medial plates. The biomechanical reasons are several. Firstly, there are reduced bending moments when using medial plates on the femur as the mechanical axis, which by definition runs from hip centre of rotation to ankle and falls slightly medial at the knee. This bending moment (load × moment arm) gives rise to the deformation; the moment arm of the load is the distance from the line of action of the load (mechanical axis) to the neutral axis of the bone and plate. The plate in turn draws the neutral axis from the centre of the femur towards the plate. Lateral plating increases the moment arm and hence increases the moment and deformation force. Conversely, with the medial plate the moment arm is smaller, and thus, deformation and plate strains would be lower. The total construct strain as recorded is much lower on the medial-sided plate, and there is no yielding. Thus, the lateral plate is more likely to undergo plastic deformation, resulting in earlier failure of the bone–plate construct. The different strain patterns recorded along different points on the plates also differed; higher strains, and thus less stability, were recorded across the wedge in the lateral plate construct.

Secondly, there is lower wedge gap displacement in a medially plated femur when loaded. Medial plating supports the deficient medial column directly and reduces the stress environment. The medial plate becomes a load-bearing cantilever implant. In vivo, it has been shown that stable fixation in the presence of medial comminution was not achievable with lateral plates as varus collapse occurred [17]. Hence, double fixation, using medial and lateral plates, was suggested. This has been linked to an increased morbidity from knee contractures and delayed union, consequent to damage to the local biology from two exposures.

Lastly, medial and lateral plates produce different mechanical environments. A lateral plate has viscoelastic properties, going from elastic to plastic behaviour as the plate spans the wedge. The medial plate, conversely, acts like a buttress on axial loading, resulting in a stiffer stronger construct. Both effects annul each other.

There are limitations in our study. A laboratory-based study makes various assumptions and inferences. Forces on the plate and fracture site are a simplification of the in vivo loads, which are a combination of axial loading, torsion and muscle pull reaction forces. Our experimental construct tested axial load only, which we believe represents the major deforming force across this fracture pattern. The medial plate is more demanding technically due to practicalities of operating on the inner side of the thigh and the close proximity of vital anatomical structures, but medial femoral sub-muscular plating is an operative technique that can be performed safely with a judicious understanding of the relevant anatomy. Lastly, synthetic sawbones were utilized in the study. Although the properties of sawbones are not identical to human bone, they have a high degree of uniformity; as such, the changes noted during testing represent true differences in the mechanical properties of the implant placement rather than differences in bone quality [18].

## Conclusion

The aim of this investigation was to determine the optimal side for plating, a comminuted supracondylar fracture of the femur. An investigation into stiffness, wedge displacement and plate strains between the lateral- and medial-sided plating was performed. The data indicate that medial-side plating is stronger and stiffer mechanically than lateral plating in this simulation.
